# Evaluation of smartphone‐based testing to generate exploratory outcome measures in a phase 1 Parkinson's disease clinical trial

**DOI:** 10.1002/mds.27376

**Published:** 2018-04-27

**Authors:** Florian Lipsmeier, Kirsten I. Taylor, Timothy Kilchenmann, Detlef Wolf, Alf Scotland, Jens Schjodt‐Eriksen, Wei‐Yi Cheng, Ignacio Fernandez‐Garcia, Juliane Siebourg‐Polster, Liping Jin, Jay Soto, Lynne Verselis, Frank Boess, Martin Koller, Michael Grundman, Andreas U. Monsch, Ronald B. Postuma, Anirvan Ghosh, Thomas Kremer, Christian Czech, Christian Gossens, Michael Lindemann

**Affiliations:** ^1^ Roche Pharma Research and Early Development, pRED Informatics, Pharmaceutical Sciences, Clinical Pharmacology, and Neuroscience, Ophthalmology, and Rare Diseases Discovery and Translational Area, Roche Innovation Center Basel, F. Hoffmann‐La Roche Ltd. Basel Switzerland; ^2^ Prothena Biosciences Inc. South San Francisco California USA; ^3^ Global R&D Partners, LLC San Diego California USA; ^4^ Felix Platter Hospital, University Center for Medicine of Aging, Memory Clinic, Basel, Switzerland; University of Basel, Faculty of Psychology Basel Switzerland; ^5^ Department of Neurology McGill University, Montreal General Hospital Montreal Quebec Canada

**Keywords:** digital health, digital biomarkers, Parkinson's disease, clinical trial, remote patient monitoring

## Abstract

**Background**: Ubiquitous digital technologies such as smartphone sensors promise to fundamentally change biomedical research and treatment monitoring in neurological diseases such as PD, creating a new domain of digital biomarkers.

**Objectives**: The present study assessed the feasibility, reliability, and validity of smartphone‐based digital biomarkers of PD in a clinical trial setting.

**Methods**: During a 6‐month, phase 1b clinical trial with 44 Parkinson participants, and an independent, 45‐day study in 35 age‐matched healthy controls, participants completed six daily motor active tests (sustained phonation, rest tremor, postural tremor, finger‐tapping, balance, and gait), then carried the smartphone during the day (passive monitoring), enabling assessment of, for example, time spent walking and sit‐to‐stand transitions by gyroscopic and accelerometer data.

**Results**: Adherence was acceptable: Patients completed active testing on average 3.5 of 7 times/week. Sensor‐based features showed moderate‐to‐excellent test‐retest reliability (average intraclass correlation coefficient = 0.84). All active and passive features significantly differentiated PD from controls with *P* < 0.005. All active test features except sustained phonation were significantly related to corresponding International Parkinson and Movement Disorder Society–Sponsored UPRDS clinical severity ratings. On passive monitoring, time spent walking had a significant (*P* = 0.005) relationship with average postural instability and gait disturbance scores. Of note, for all smartphone active and passive features except postural tremor, the monitoring procedure detected abnormalities even in those Parkinson participants scored as having no signs in the corresponding International Parkinson and Movement Disorder Society–Sponsored UPRDS items at the site visit.

**Conclusions**: These findings demonstrate the feasibility of smartphone‐based digital biomarkers and indicate that smartphone‐sensor technologies provide reliable, valid, clinically meaningful, and highly sensitive phenotypic data in Parkinson's disease. © 2018 The Authors. Movement Disorders published by Wiley Periodicals, Inc. on behalf of International Parkinson and Movement Disorder Society.

There is a growing anticipation that digital telemedicine technologies that measure Parkinson's disease (PD) signs will revolutionize clinical research and treatment monitoring.^1^ These tools can provide frequent and mobile measurement of clinically relevant signals using electronic sensors that combine active (i.e., performing specific testing protocols) and passive protocols (i.e., monitoring movement during daily life), to quantify and/or predict health‐related outcomes and support diagnostic processes. This approach promises to modernize clinical trial endpoints, improve clinical study design, and advance treatment monitoring.^2^, ^3^

Digital biomarkers offer three main advantages compared with status quo in‐clinic testing. First, by using objective multivariate sensor data with apps designed to measure clinical signs and symptoms, they can potentially quantify symptom severity with greater sensitivity and objectivity than clinical rating scales.^4^ Second, with daily active tests and continuous passive monitoring, they enable even daily testing throughout longitudinal studies, a test frequency that was unthinkable just a few years ago. This promises greater measurement accuracy compared with data collected from clinic visits spaced weeks or months apart. This is particularly key for diseases with variable symptom severity such as PD. Last, with passive monitoring, the effects of PD on patients' daily lives can now be monitored and quantified in their usual home settings,^5^ providing ecologically valid metrics to assess disease status and treatment effects.

Although numerous commercial digital biomarker devices are available,^6^, ^7^, ^8^ common consumer technologies such as smartphones have the advantages of widespread availability, low cost, and high sensor quality. Several pilot research studies have successfully developed smartphone applications for PD.^1^, ^9^, ^10^, ^11^, ^12^, ^13^, ^14^, ^15^, ^16^, ^17^ In these pilot studies, proof of concept was typically established in a clinical setting by demonstrating significant differences between individuals with PD and healthy controls, and/or significant relationships between the sensor‐based measures and the International Parkinson and Movement Disorder Society–Sponsored Revision of the Unified Parkinson's Disease Rating Scale (MDS‐UPDRS) clinical gold standard.^14^, ^18^, ^19^, ^20^, ^21^, ^22^, ^23^, ^24^ For example, Kassavetis and colleagues^18^ tested 14 PD participants (mean disease duration = 3.7 years) with the MDS‐UPDRS^25^ and a custom Android application with the following active tests: resting, postural and kinetic tremor, pronation‐supination, leg agility, and finger‐tapping. For all tasks, the extracted sensor feature data significantly correlated with corresponding MDS‐UPDRS^25^ item scores (e.g., item 3.17, rest tremor amplitude).

Remote deployment of digital biomarker testing suites has been reported in far fewer studies. Arora and colleagues^19^ deployed an Android smartphone to 10 individuals with PD (mean motor MDS‐UPDRS score = 19.6 [standard deviation {SD} = 6.7]) and 10 controls, who performed four active tests (sustained phonation, gait, finger‐tapping, and reaction time) four times daily for 1 month. Adherence was acceptable at 69%, and random forest machine‐learning successfully discriminated PD participants from controls.^27^ Using a similar approach on iPhones (Apple Inc., Cupertino, CA), the mPower app was launched with Apple's Research Kit platform (Apple Inc.) in March 2015^28^, ^29^ with surveys and the same tasks developed by Arora and colleagues.^19^ In this study, 1,087 self‐declared persons with PD and 5,581 self‐declared persons without PD completed at least one active test or survey.^28^ Adherence dropped sharply after the first few days post‐download; 898 individuals contributed ≥ 5 days' data during the first 6 months following download. Data analysis of these results is pending. The Parkinson@home study^26^, ^30^ deployed the Fox Wearable Companion app to 953 participants in the Netherlands (13‐week study) and North America (6‐week study) to passively monitor behavior and rate the severity of selected symptoms. Adherence was 68% in the Netherlands and 62% in North America, and not affected by demographics, clinical characteristics, or attitude toward technology.^26^, ^30^ Further data analyses are pending. These preliminary findings represent major milestones in the development of digital biomarkers, and at the same time highlight the challenges yet to be overcome for the successful use of digital biomarker approaches in clinical research or patient monitoring, namely long‐term adherence, consistency of sensor‐derived feature data, and clinical validity of long‐term, remote patient testing.

The present study, which began in September 2015, determined the feasibility, reliability, and validity of digital biomarkers in a 6‐month clinical trial of individuals with PD. These findings will provide a foundation for the future use of digital biomarkers in clinical trials and treatment monitoring of PD.

## Materials and Methods

We report data from two independent smartphone‐based remote monitoring studies: a 6‐month, phase 1b clinical drug trial of RG7935/PRX002 with 44 PD participants (NCT02157714); and a 6‐week observational study of 35 age‐ and sex‐matched controls. The effects of RG7935/PRX002 are not the focus of this study and are not reported here. Both studies were approved by the respective local ethics committees and written, informed consent was obtained from all participants (patient study: IRB00010809, H‐35018, WOR1‐14‐143; control study: EKNZ‐BASEC‐2016‐00596).

### Participants

Forty‐four individuals with PD participated. Data from 1 patient was lost because of a lost phone. Thirty‐five age‐/sex‐matched healthy controls were recruited from the Registry of Research‐Interested Healthy Individuals at the Memory Clinic, University Center for Medicine of Aging Basel, Felix‐Platter Hospital, Switzerland. All controls scored ≥ 26 points on the Montreal Cognitive Assessment (MoCA) 32 and were free of cardiovascular, neurological, or psychiatric conditions and had no first‐degree relative with PD. The participants' baseline characteristics (including PD subtype^33^) are described in Table 1.

**Table 1:** Baseline demographic and clinical characteristics of the PD and control participants

Characteristic	PD	Controls	Difference (*P* Value)
N	43	35	
Age (yr)	57.5 ± 8.45	56.23 ± 7.83	0.5
Male Female	35 8	27 8	0.64
H & Y stage	1.91 ± 0.48	n/a	
Total MDS‐UPDRS^a^	45.41 ± 17.22	3.17 ± 2.7	<0.001
MDS‐UPDRS I	6.91 ± 4.71	1.80 ± 2.08	<0.001
MDS‐UPDRS II	9.21 ± 6.10	0.23 ± 0.73	<0.001
MDS‐UPDRS III	27.67 ± 11.22	1.14 ± 1.06	<0.001
MDS‐UPDRS IV	1.63 ± 2.65	0 ± 0	<0.001
MoCA	26.86 ± 2.46	28.34 ± 1.35	0.001
Mean disease duration (yr)	3.51 ± 2.86	n/a	
Proportion of patients taking dopaminergic medication	81%	n/a	
Experiencing fluctuations (%)^b^	37% (30% experience at least a slight impact)	n/a	
MDS‐UPDRS‐defined TD and PIGD PD subtypes	29 TD; 9 PIGD; 5 indeterminate	n/a	
Experiencing dyskinesia (%)^c^	9.3%	n/a	

Data are mean ± SD or proportions.

a Total MDS‐UPDRS = MDS‐UPDRS I + MDS‐UPDSR II + MDS‐UPDRS III.

b Proportion of patients with MDS‐UPDRS 4.3 “time spent in the off state >0.”

c Proportion of patients with MDS‐UPDRS 4.1 “time spent with dyskinesia >0.” A total of 1 PD had functional impact of dyskinesias, indicated by MDS‐UPDRS 4.2 > 0.

TD, tremor dominant. n/a, not applicable.

### MDS‐UPDRS

The MDS‐UPDRS^25^ (defined as gold standard for validation) was administered by trained raters (parts I and II) and physicians (part III) to PD participants during screening (study day −42 to −1) and days 8 and 64. Trained raters tested controls at baseline and day 42.

### Smartphone Testing

Procedures were identical for the PD and control studies. At initial in‐clinic visits, all participants received a smartphone (Galaxy S3 mini; Samsung, Seoul, South Korea) with the Roche PD Mobile Application v1 (Roche, Basel, Switzerland) preinstalled, and a belt containing a pouch in which to carry the phone. Smartphones were locked‐down (i.e., configured so patients could only run the Roche PD Mobile Application v1 and WiFi connection software). Training on the active tests was provided by site staff. Participants were instructed to complete the active tests at home once daily (ideally in the morning) and carry the phone with them throughout the day, recharging the phone overnight. The time of patients' medication intake was not consistently collected. Battery charge lasted on average 7 hours per day.

### Active Tests

**Figure 1:**
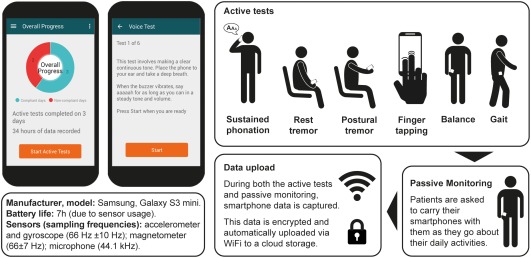
Screenshots of the smartphone application and workflow for the daily assessments. The smartphone (Galaxy S3 mini; Samsung, Seoul, South Korea) was provided with a single, preinstalled custom application (Roche PD Mobile Application v1; Roche, Basel, Switzerland). The application requested the completion of six active tests daily and subsequently recorded sensor data during daily living (“passive monitoring”), whereby participants were instructed to carry the smartphone in their trouser pocket, or a small bag around the waist.

An Android custom application (Roche PD Mobile Application v1) was designed to measure cardinal PD motor signs (tremor, bradykinesia, and rigidity/postural instability) with inertial measurement unit sensor data (i.e., accelerometer, gyroscope, and magnetometer) and voice recorded with microphone (see Fig. 1). The app included the following six active tests (see Supplementary Data available online for detailed instructions).

1. Sustained phonation: making a continuous “ahh” sound for as long as possible.
2. Rest tremor: seated, holding the phone in the palm of the hand resting on the lap.
3. Postural tremor: seated, holding the phone in their outstretched hand.
4. Finger‐tapping: with the smartphone on a flat surface (e.g., table) alternately tapping two touchscreen buttons as regularly (i.e., not as quickly) as possible.
5. Balance task: standing still while having the smartphone in the trouser pocket or a specially‐designed belt pouch.
6. Gait task: with the smartphone in the trouser pocket/belt pouch, walking 20 yards, turning around, and returning to the starting point.

Each task was preceded by a screen which named and explained the task. Participants pressed a button to start the task, after which a brief countdown appeared, then a start tone, followed by 30 seconds for the task concluding with a tone that signaled the end of the task. Participants were instructed to go outside to complete the gait task if they had no unobstructed 20‐yard path to walk in their homes. The sustained phonation task recorded from the built‐in microphone, and the finger‐tapping test recorded all touchscreen events.

### Passive Monitoring

Participants were instructed to carry the phone with them throughout the day (e.g., in the trouser pocket or provided belt pouch) enabling passive monitoring of daily activity via smartphone sensors.

### Data Transfer

The inertial measurement unit sensors sampled nonuniformly (accelerometer and gyroscope: 66 ± 10 Hz; magnetometer: 66 ± 7 Hz; microphone: 44.1 kHz), with comparable averages and SDs across phones. These time‐stamped data were collected continuously while the smartphone battery was active (i.e., during active tests and passive monitoring). Participants received instructions on how to connect the smartphone to the Internet at home. If their home had no Internet, data were uploaded during site visits. All data were stored in encrypted files on the smartphone and sent by WiFi to a cloud storage facility each time the smartphone connected to the Internet.

### Data Processing

Data underwent quality control to ensure usability (e.g., sufficiently long sustained phonation; no walking during balance test). This resulted in the removal of 15% of sustained phonation data and 3% of all other active test data. Comparable amounts of data were discarded from PD and controls for each test. The following active test features were selected, based on previous literature and their relevancy to PD: (1) Sustained phonation: mel‐frequency cepstral coefficient 2 (MFCC2) 34, 35; (2) Rest tremor: skewness^36^, ^37^, ^38^, ^39^; (3) Postural tremor: total power^40^, ^41^; (4) Finger tapping: intratap variability^18^, ^42^; (5) Balance: mean velocity^43^, ^44^, ^45^, ^46^, ^47^; and (6) Gait: turn speed^46^, ^48^, ^49^, ^50^ (Table 2; see Supplementary Data available online and Cheng and colleagues^51^ for additional details). For PD participants, feature data were averaged over the 2‐week period corresponding to the weeks before and after the site visits with the MDS‐UPDRS. For controls, feature data were averaged over the first 2 study weeks and compared to baseline MDS‐UPDRS.

**Table 2:** Smartphone sensor features from each active and passive monitoring activity and corresponding MDS‐UPDRS item(s) score, associated test‐retest reliability, ability to detect control vs. PD differences, relationship with clinical symptom severity and ability to detect signs in PD participants clinically scored as having no signs

Activity	Sensor Feature and Unit	MDS‐UPDRS Item(s)	Test‐Retest Reliability of Sensor Feature	Controls vs. All PD		PD Participants: Relationship Between Active Test Feature and MDS‐UPDRS Item(s) Score		Controls vs. PD Subgroup Rated as Having No Symptoms (MDS‐UPDRS score = “0”) at Respective Site Visit	
Active tests			ICC	*t* Value	*P* Value	*t* Value	*P* Value	*t* Value	*P* Value
Sustained phonation	MFCC2	2.1	0.82	−4.48	<0.001	–0.36	n.s	−3.29	<0.001
*Rest tremor*	Skewness	3.18	0.90	7.6	<0.001	2.17	0.033	5.5	<0.001
Postural tremor	Power (m^2^/s^3^)	2.10	0.97	4.42	<0.001	2.61	0.011	0.83	n.s.
Finger tapping	Tap variability	2.5	0.64	8.46	<0.001	2.18	0.028	6.95	<0.001
Balance	Mean velocity (m/s)	3.13	0.80	8.29	<0.001	2.38	0.027	5.16	0.005
Gait	Turn speed (degree/s)	PIGD	0.88	−9.31	<0.001	−2.45	0.022	−8.25	<0.001
Passive monitoring			ICC	Mann‐Whitney U	*P* Value	*t* Value	*P* Value	Mann‐Whitney U	*P* Value
Walking	Turn speed (degree/s)	Average PIGD	n/a	1,392	<0.001	−3.01	0.005	229	0.018
Sit‐to‐stand transitions	Sit‐to‐stand transitions (#/hour)	Average PIGD	n/a	1,087	0.005	−1.48	n.s.	250	n.s.
Walking vs. not walking	Activity ratio (%)	Average PIGD	n/a	1,392	<0.001	−2.02	0.055	321	0.003

Values for the active tests are *t* values from linear mixed‐effects models that quantify the relationship between given MDS‐UPDRS item(s) and the selected features and take repeated tests over time of the same subjects into account. Values for the passive monitoring activities are U values from the Mann‐Whitney U test.

MFCC2, mel‐frequency cepstral coefficient 2; n/a, not applicable; n.s., not significant; STS, sit‐to‐stand/stand‐to‐sit transitions.

A machine‐learning algorithm based on a Human Activity Recognition (HAR) model^52^ was used to classify passive monitoring accelerometer data into labeled activities (e.g., walking, climbing stairs, standing, and sitting).^50^

The HAR model was trained on two independent public data sets of everyday activity from normal individuals^53^, ^54^; of these data, 90% were used to train the HAR model and 10% for model validation (see Cheng and colleagues^51^ for details). To test the model's robustness and its appropriateness for the PD sample, two independent validations were performed: First, the model was tested on the validation data sample, where it reached 98% accuracy in distinguishing stationary from gait activities; second, the model's accuracy was tested on the unlabeled PD active test data, where it reached 99.5% and 96.9% accuracy when classifying PD balance and gait active test data, respectively. The HAR model was then applied to label human activities using a 5‐second moving average, from which the following features were extracted: activity ratio^50^, ^55^, ^56^, ^57^ (i.e., time walking divided by total passive monitoring time), sit‐to‐stand transitions,^45^, ^50^, ^58^ and turn speed^46^, ^48^, ^49^, ^50^, ^59^, ^60^ (Table 2).^50^ One averaged summary score for each feature and participant was calculated over the whole observation period.

### Statistical Analyses

Adherence was quantified as the percentage of active tests completed for each study week (maximum: seven tests completed in 7 days; 100% adherence) per participant. Test‐retest reliability of active test feature data from PD participants was assessed using the intraclass correlation coefficient (ICC) between the first and second 14‐day testing periods.^61^ Because of their non‐normal distributions, Mann–Whitney U tests were used to compare passive monitoring features between PD and control participants.

Clinical validity of active test features was tested by relating each feature score, averaged over 2 weeks, to the corresponding MDS‐UPDRS^25^ item score (Table 1, columns 1‐2 and 3). Thus, for each MDS‐UPDRS item or subscale score for each patient, we compared an active test feature score comprised of the average performance estimate from all active test sessions conducted within the 2‐week period surrounding (during the trial) or adjacent to (beginning and end of trial) the site visit at which the MDS‐UPDRS was administered. The postural instability and gait difficulty (PIGD) 33 subscore (i.e., MDS‐UPDRS 2.12 + 2.13 + 3.10 + 3.11 + 3.12) was selected as the comparator for the turn speed feature. No direct comparator was available for the sustained phonation feature; therefore, speech (item 2.1) was used as the closest correlate. Linear mixed‐effects models tested for significant relationships between each active test feature and the corresponding MDS‐UPDRS item score, with individuals as random effects (to control for repeated MDS‐UPDRS measurements per subject) and sex and age as covariates. Homoscedasticity was evident for all models. We report *t* values and associated *P* values from the models to quantify these relationships.

The ability of each active test feature to detect PD signs was tested by comparing feature scores between controls and all PD participants. Moreover, the ability to detect subtle or variable PD signs was tested by comparing features scores between controls and those PD participants whose corresponding MDS‐UPDRS score or PIGD subscale score was 0 (Table 2, column 7). These analyses used linear mixed‐effects models which covaried age and sex. Homoscedasticity was apparent for all models.

## Results

### Adherence

PD participants completed a total of 5,135 active tests, adhering to 61% of all possible test sessions during the 6‐month trial period. Sixty‐four percent of PD participants completed all active tests at least once every other day and 90% at least once every 4 days. Even in the last week of the 6‐month study, PD participants completed all tests on average 3 of 7 days per week. A total of 1,542 active tests were completed by controls during their 6‐week study, corresponding to an overall adherence of 100% in the shorter protocol. A total of 24,104 hours of passive monitoring data was collected from PD participants and 8,614 hours from controls.

### Reliability of Testing

Active test features demonstrated “excellent” (postural tremor) or “moderate to good” (all remaining features) 62 test‐retest reliability (ICC; Table 2).

### Clinical Validity

**Figure 2:**
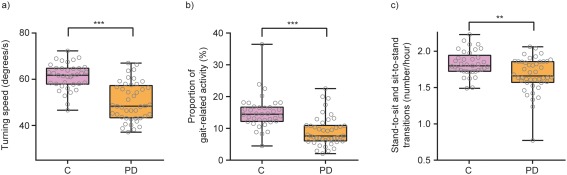
Machine‐learning algorithms applied to passive monitoring data revealed multiple aspects of significantly reduced everyday motor behavior in PD participants compared with controls. See Results (Reliability of Testing) for details. ^**^ *P* < 0.01; ^***^ *P* < 0.001. C, control group.

Clinical validity of the active and passive sensor features was first evaluated by testing their ability to discriminate PD participants from controls. All active test and passive monitoring features significantly discriminated PD from controls (all *P* < 0.005; Table 2). Compared to controls, PD participants manifested 34% less time performing gait‐related activities (PD = 0.1 ± 0.05, controls = 0.15 ± 0.05 proportion of time spent walking), 8.2% fewer sit‐to‐stand transitions (PD = 1.67 ± 0.23, controls = 1.82 ± 0.17 per hour) and 18% slower > 90‐degree turns (PD = 50.3 ± 8.2, controls = 61.2 ± 5.6). Single participant data are shown in Figure 2, where each data point represents motor behavior averaged over the duration of the study, summarized with boxplots.

**Figure 3:**
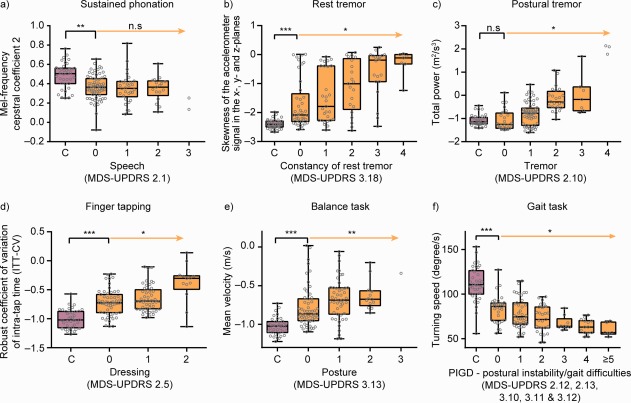
Active test feature scores aggregated over 2 weeks of in‐home testing demonstrated case‐control differences, significant relationships with clinical severity ratings, and significantly greater sensitivity compared with MDS‐UPDRS item/subscale scores from site visits. The orange arrow indicates the statistical test for association of increased disease severity as to the selected MDS‐UPDRS item with the digital biomarker feature, taking into account repeated measures per participant. The black square bracket indicates a comparison of the control group (C) with PD participants that are rated “0” for the corresponding MDS‐UPDRS item. ^*^ *P* < 0.05; ^**^ *P* < 0.01; ^***^ *P* < 0.001. C, control group; MFCC2, mel‐frequency cepstral coefficient 2; n.s, not significant.

Clinical validity of the active test features was further assessed by comparing smartphone‐based estimates of symptom severity (i.e., feature values) with corresponding MDS‐UPDRS item or subscale scores (Fig. 3). These linear‐effects mixed models revealed that all active test features, with the exception of sustained phonation, were significantly related to their corresponding MDS‐UDPRS scores (Table 2; Fig. 3). For the passive monitoring features, only turn speed was significantly related to average PIGD scores; both sit‐to‐stand transitions and activity ratio showed trends for a relationship with average PIGD (Table 2).

### Sensitivity for Undetected Manifestations

The ability of the active and passive test features to detect subtle or variable PD signs/symptoms was assessed through comparison of the feature scores of a subset of PD participants scored as having no signs/symptoms on the respective MDS‐UPDRS item at the site visit (i.e., with a MDS‐UPDRS item score of 0) with controls. These analyses revealed that all active test features except postural tremor, and sit‐to‐stand transitions and activity ratio passive monitoring measures, detected significant PD‐like abnormalities (compared with controls) in PD participants who demonstrated no signs on the corresponding MDS‐UPDRS item(s) (Table 2; Fig. 3). It is important to note that postural tremor sensor measures were compared to patients' self‐report of tremor interfering with daily life (MDS‐UPDRS item 2.10); in this context, the negative result for postural tremor indicates that the sensor feature indeed accurately reflected patients' self‐perceived symptom severity.

### Discussion

The present study is, to our knowledge, the first reported deployment of a digital biomarker approach in a clinical trial of PD. It provides an initial demonstration that use of commercially available smartphones for in‐home active testing and passive monitoring in the home environment is feasible and provides reliable, clinically meaningful, and sensitive symptomatic data. All active and passive test features significantly differentiated controls from PD participants, and the magnitude of sensor‐based features was significantly related to physician‐administered MDS‐UPDRS scores. Passive monitoring data revealed significantly reduced mobility in PD participants compared to controls. Finally, active tests could detect significant abnormalities even in PD participants who were rated as having no evidence of the corresponding sign on exam, suggesting that digital biomarkers may confer important sensitivity advantages. Together, these results indicate that a digital biomarker approach fulfills the prerequisites of acceptable adherence, reliability, validity, and sensitivity for use in long‐term clinical trials and treatment monitoring.

The current protocol instructed PD participants to complete six daily active assessments over a period of 6 months. At the outset, it was unclear whether PD participants would adhere to this testing schedule (the testing procedure itself is short, approximately 5 minutes). We found that overall adherence to the active test protocol was 61% over 6 months, similar to a smaller‐scale digital biomarker approach with four active tests performed four times daily for 1 month (69%^19^), and greater than what was observed with the general population deployment of mPower.^28^ This demonstration of long‐term adherence passes a crucial test for future digital biomarker use in long‐term clinical trials and eventually for patient monitoring.

Beyond feasibility, the digital biomarker approach must produce sensor feature data of adequate reliability and validity to be used in research and clinical settings. Reliability analyses confirmed this to be the case: ICCs ranged from moderate (finger tapping; 0.64) to excellent (postural tremor: 0.97) for all active tests. The clinical validity of the active and passive test features were confirmed in two independent sets of analyses. First, all sensor features exhibited clear disease‐relevant signals by significantly differentiating PD participants from controls. Second, all but one sensor feature demonstrated a significant relationship between severity as estimated by the sensors and as rated by the physician. The exception was sustained phonation, where the MFCC2 feature was not significantly related to MDS‐UPDRS Speech, item 2.1.^25^ However, note that MFCC2 reflects the ratio between vocal tract resonation of the high, and vocal fold vibration of the low, Mel‐frequency bands^67^; a parameter not specifically scored by MDS‐UPDRS item 2.1.^25^

Daily sensor feature data appeared to be more sensitive at detecting subtle abnormalities than the less frequent physician ratings. Of note, significant motor impairments (compared to controls) were observed even in those PD participants rated as having no motor impairment on the corresponding MDS‐UPDRS item(s) on five of the six feature variables (i.e., sustained phonation, resting tremor, finger tapping, walking speed, and turning speed). Only one (postural tremor) feature failed to detect this. However, this corresponding MDS‐UPDRS item assessed the *self‐perceived* interference of tremors on patients' daily functioning (and not postural tremor on examination). Patients who negated this phenomenon had sensor postural tremor scores on par with controls, thus indicating that the sensor accurately recapitulated patients' self‐perception of tremor severity. For all MDS‐UPDRS item(s) based on physician's ratings, the digital biomarker feature showed greater sensitivity, suggesting that a primary reason for the improved sensitivity is frequency of sampling. Clearly, frequent (i.e., daily) smartphone‐based active testing has the advantage of sampling many more data points, increasing power to detect potentially subtle or variable PD motor signs, compared with much less frequent and time‐limited clinical assessment. Moreover, PD participants' motor signs may be less severe in the activating presence of a physician than during daily life at home, leading to an underestimation of motor severity at clinic visits.^68^ Taken together, these results indicate that a frequent and remote digital biomarker approach is more sensitive at detecting subtle and potentially variable motor impairments than MDS‐UPDRS ratings at infrequent site visits.

There is a discontinuous mapping between motor performance during active, maximum capacity testing and patients' functional capacity in daily life.^69^ For this reason, motor behavioral data acquired from continuous passive monitoring while patients go about their daily lives represent a critical complement to the clinical picture obtained from active or in‐clinic testing. The machine‐learning algorithms applied to passive monitoring generated a HAR model which revealed that PD participants, even those scored as having no gait dysfunction at the site visits, were significantly less active than controls in several aspects of daily living: time spent performing gait‐related activities, sit‐stand transitions, and turn speed. Note that a cultural confound may partially explain these findings given that controls were recruited from Switzerland and PD participants from the United States. Critically, however, evaluating the PD sample alone revealed that turn speed was significantly related to PIGD scores, and gait activity showed a trend relationship with PIGD. The present findings are also in line with other studies measuring remote physical activity in PD, which were traditionally performed over shorter timeframes (i.e., 1 day to 1 week). These reported significantly less motor activity in PD compared to controls (e.g., fewer steps and shorter bouts of physical activity,^48^, ^70^) some of which were related to disease severity^71^ (for a review, see Block and colleagues^5^). Such insights may carry significant weight when assessing drug efficacy; that is, the ability of the therapy to improve patients' ability to perform everyday activities, which may be indirectly related to quality of life.^72^, ^73^

Several limitations to this study exist. First, we did not control for participants' familiarity with digital technology including smartphones, which may affect active test performance or passive monitoring adherence. Second, note that ICCs calculated with mean data may lead to artificially heightened ICC values compared with ICCs calculated using individual data points. Third, whereas the applicability of our HAR model (generated using control data) to PD patient data was confirmed through successful categorization of PD active test data (i.e., data where activities being performed were known), feature extraction from PD passive monitoring may be improved through HAR models generated with combined control and patient data. In addition to these points, future research including the determination of appropriate feature cut‐off values for healthy/diseased classifications, and prospective assessments to determine longitudinal relationships between digital biomarker features and clinical measures is warranted.

The promise of digital biomarkers to create a paradigm shift in biomedical research and clinical treatment monitoring is evident.^3^ Our study demonstrates that digital biomarkers have now become feasible. Future research is needed to confirm whether digital biomarkers provide valid and fine‐grained measures of disease severity and treatment response, enabling a better understanding of our patients' daily lives.

## Author Roles

(1) Research Project: A. Conception, B. Organization, C. Execution; (2) Statistical Analysis: A. Design, B. Execution, C. Review and Critique; (3) Manuscript Preparation: A. Writing of the First Draft, B. Review and Critique.

F.L.: 1B, 1C, 2A, 2B, 2C, 3A, 3B

K.I.T.: 1B, 1C, 2C, 3A, 3B

T.K.: 1B, 1C, 3B

D.W.: 1C, 3B

A.S.: 1B, 1C, 2A, 2B, 2C, 3B

J.S.‐E.: 1B, 1C, 3B

W.‐Y.C.: 2A, 2B, 2C, 3B

I.F.‐G.: 1B, 1C, 2A, 2B, 2C, 3B

J.S.‐P.: 2A, 2B, 2C, 3B

L.J.: 1B, 1C, 3B

J.S.: 1B, 1C, 2C, 3B

L.V.: 1B, 1C, 3B

F.B.: 1B, 1C, 2C, 3B

M.K.: 2C, 3B

M.G.: 1B, 1C, 2C, 3B

A.U.M.: 1B, 1C, 2C, 3B

R.B.P.: 2C, 3B

A.G.: 1A, 1B, 3B

T.K.: 1A, 1B, 3B

C.C.: 1A, 1B, 1C, 3B

C.G.: 1A, 1B, 1C, 2C, 3B

M.L.: 1A, 1B, 1C, 2A, 2B, 2C, 3A, 3B

## Financial Disclosures

Florian Lipsmeier: Employee, F. Hoffmann‐La Roche AG, Basel, Switzerland. Kirsten I. Taylor: Employee, F. Hoffmann‐La Roche AG, Basel, Switzerland. Timothy Kilchenmann: Employee, Inovigate (Schweiz) GmbH, Basel, Switzerland. Detlef Wolf: Employee, F. Hoffmann‐La Roche AG, Basel, Switzerland. Alf Scotland: Employee, Inovigate (Schweiz) GmbH, Basel, Switzerland. Jens Schjodt‐Eriksen: Employee, NNIT, Zurich, Switzerland. Wei‐Yi Cheng: Employee, Roche TCRC Inc, New York, New York, USA. Ignacio Fernandez‐Garcia: Employee, F. Hoffmann‐La Roche AG, Basel, Switzerland. Juliane Siebourg‐Polster: Employee, F. Hoffmann‐La Roche AG, Basel, Switzerland. Liping Jin: Employee, Roche TCRC Inc, New York, New York, USA. Jay Soto: Employee and stockholder, Prothena Biosciences, San Francisco, California, USA. Lynne Verselis: Employee, Roche TCRC Inc, New York, New York, USA. Frank Boess: Employee, F. Hoffmann‐La Roche AG, Basel, Switzerland. Martin Koller: Employee and stockholder, Prothena Biosciences, San Francisco, California, USA. Michael Grundman: Consultant to Prothena Biosciences, San Francisco, California, USA. Andreas U. Monsch: Honoraria from Roche, Vitor; Advisory Board attendance for AC Immuna, Roche, Vifor, Merck Sharp & Dohme, AbbVie; and grants from Roche, Vifor. Ronald B. Postuma: Personal compensation for travel, speaker fees, and consultation from Boehringer Ingelheim, GE Health Care, Novartis, Roche, Theranexus, and Teva Neurosciences; is funded by grants from the Fonds de Recherche du Quebec ‐Sante, the Michael J. Fox Foundation, the W. Garfield Weston Foundation, and the Canadian Institutes of Health Research. Anirvan Ghosh: Employee and stockholder, Biogen, Cambridge, Massachusetts, USA. Thomas Kremer: Employee, F. Hoffmann‐La Roche AG, Basel, Switzerland. Christian Czech: Employee and stockholder, F. Hoffmann‐La Roche AG, Basel, Switzerland. Christian Gossens: Employee and stockholder, F. Hoffmann‐La Roche AG, Basel, Switzerland. Michael Lindemann: Professorship, Baden‐Wuerttemberg Cooperative State University, Germany.

## Supporting information

Supplementary Information 1
