# Supplementary material for: Evaluation of smartphone‐based testing to generate exploratory outcome measures in a phase 1 Parkinson's disease clinical trial
Source: Mov Disord. 2018 Apr 27;33(8):1287–97. doi: 10.1002/mds.27376 (PMC6175318; doi:10.1002/mds.27376)
Supplement: Supplementary file 1 — Supplementary Information 1 [file MDS-33-1287-s001.docx]

**Supplemental data to:**

**Exploratory outcome measures from smartphone-based testing in a phase 1 Parkinson’s disease trial**

Florian Lipsmeier, Kirsten I. Taylor, Timothy Kilchenmann, Detlef Wolf, Alf Scotland, Jens Schjodt-Eriksen, Wei-Yi Cheng, Ignacio Fernandez Garcia, Juliane Siebourg-Polster, Liping Jin, Jay Soto, Lynne Verselis, Frank Boess, Martin Koller, Michael Grundman, Andreas U. Monsch, Ronald B. Postuma, Anirvan Ghosh, Thomas Kremer, Christian Czech, Christian Gossens, and Michael Lindemann

# Test instructions, and active test feature and MDS-UPDRS item selection

Active test features and corresponding MDS-UPDRS items were selected according to the following guiding principles: features from a given test should be solidly anchored in the literature and physiologically interpretable in the context of Parkinson’s disease (PD); selected features should provide additional insights into symptom severity measurement via smartphone technology; corresponding MDS-UPDRS items should be related to the information gained from a given smartphone test; and over the entire active testing suite, MDS-UPDRS comparators from both Parts II and III should be represented.

Below, we provide the exact instructions for each test that were provided to patients via the app, whereby a tone and vibration indicated the beginning and end of each 30-second test. In addition, the justification for the selection of both the specific sensor feature(s) and corresponding MDS-UPDRS item(s) in each active test are provided.

### Sustained phonation

Instructions: “*This test involves making a continuous ‘aaaah’ sound in a steady pitch and volume for as long as you can. Place the phone to your ear and take a deep breath.”*

The mel-frequency cepstral coefficient 2 (MFCC2), the ratio between vocal tract resonation of the high and vocal fold vibration of the low Mel-frequency bands,^1^ was selected as the sustained phonation feature on the basis of its previous use in the literature.e.g.^2, 3^

Only two MDS-UPDRS items are related to the sustained phonation task, MDS-UPRDS items 2.1 (self-rated speech) and 3.1 (physician-rated speech). Of these, MDS-UPDRS item 2.1. was selected for comparison with MFCC2 as it was hypothesized that patients’ assessments of their perceived speech difficulties were more closely related to MFCC2 than physicians’ assessments of patients’ overall speech quality.

### Rest tremor

Instructions: “*Sit upright and hold the phone in the hand that is most affected by tremor. Rest this hand lightly in your lap, close your eyes and count backwards from 100 when the buzzer sounds. Stop at the 2^nd^ buzzer.”*

Skewness of the accelerometer signal in the x-, y- and z-planes was selected as the feature for rest tremor following previous research.^4-7^ Skewness reflects the constancy of rest tremor during each 30-second measurement interval.

MDS-UPDRS item 3.18, constancy of rest tremor, was selected as the comparator for skewness.

### Postural Tremor

Instructions: “*Keep holding the phone in the hand most affected by tremor. Hold the phone in front of you with your arm outstretched and the palm of your hand facing up.”*

Total power of the accelerometer was selected as the feature for the postural tremor test,^8, 9^ as previous research demonstrates that it describes the total amount of energy expended by movements of different parts of the body whilst the phone is held in an outstretched hand.

MDS-UPDRS item 2.10 (self-assessed tremor) was selected as the corresponding MDS-UPDRS item as it was hypothesized that this rating, encompassing patients’ perceived global tremor severity in their everyday lives, was most closely related to total power during the daily in-home postural tremor active tests.

### Finger tapping

Instructions: “*With the phone on a flat surface, tap the buttons with your index and middle finger. Tap the buttons, one after the other in a regular rhythm, until the buttons disappear.”*

We chose tapping variability^10, 11^as a feature because it exemplifies our ability to use sensor data to measure physical symptoms that are not easily accessible without high resolution measurements. The variability in the amount of time a finger spends on the smartphone screen for each tap reflects fine motor skills difficult to assess with the naked human eye.

Several dexterity-related MDS-UPDRS items are associated with the finger tapping test: items 2.4 (eating task), 2.5 (dressing), 2.7 (handwriting), 3.4 (finger tapping) and 3.5 (hand movements). MDS-UPDRS 3.4 appears most closely related, however the smartphone test did not enforce the standard clinical tapping rules to tap large and wide for 5-10 seconds. Therefore, important aspects of item 3.4 were not recapitulated in the smartphone tapping test. (Indeed, tapping variability was not significantly associated with MDS-UPDRS item 3.4 scores (t = 1.1844, p = 0.2403).) MDS-UPDRS item 2.5 was selected as the comparator because it estimates patients’ daily problems with dressing, a task requiring fine motor skills and therefore conceptually similar to the ability to tap regularly on a screen.

### Balance

Instructions: “*Stand upright and without support, with the phone in your pocket (or in a fanny pack). When the buzzer vibrates, stand unaided until the buzzer vibrates a second time.”*

Mean velocity of the accelerometer was selected as the feature for the balance test as it is well-established in the literature.^12-16^

MDS-UPDRS item 3.13 (posture) was selected as the comparator as it did not encompass additional actions (e.g. items 2.12 walking and balance, 3.9 arising from a chair measure other motor behaviors in addition to balance).

### Gait

Instructions: “*Find a place to walk for about 20 yards, turn around and return to your start position. Place the phone close to your hip (in pocket or fanny pack) and walk when the buzzer vibrates. Continue until the buzzer vibrates again.”*

Turn speed was selected as the most relevant feature in the gait task,^15, 17-19^ as turning is a movement susceptible to difficulties in both gait and balance.

The postural instability and gait difficulty (PIGD) subscore^20^ (a composite of MDS-UPDRS items 2.12, 2.13, 3.10, 3.11 and 3.12) was selected for comparison with the turn speed feature as it captures the information most relevant to gait in the MDS-UPDRS.

## References

1. Koo TK, Li MY. A Guideline of Selecting and Reporting Intraclass Correlation Coefficients for Reliability Research. J Chiropr Med 2016; 15: 155–163.

2. Salarian A, Russmann H, Wider C, Burkhard PR, Vingerhoets FJ, Aminian K. Quantification of tremor and bradykinesia in Parkinson's disease using a novel ambulatory monitoring system. IEEE Trans Biomed Eng 2007; 54: 313–322.

3. Taylor Tavares AL, Jefferis GS, Koop M, et al. Quantitative measurements of alternating finger tapping in Parkinson's disease correlate with UPDRS motor disability and reveal the improvement in fine motor control from medication and deep brain stimulation. Mov Disord 2005; 20: 1286-1298.

4. Ossig C, Antonini A, Buhmann C, et al. Wearable sensor-based objective assessment of motor symptoms in Parkinson's disease. J Neural Transm (Vienna) 2016; 123: 57–64.

5. Broen MP, Marsman VA, Kuijf ML, Van Oostenbrugge RJ, van Os J, Leentjens AF. Unraveling the Relationship between Motor Symptoms, Affective States and Contextual Factors in Parkinson's Disease: A Feasibility Study of the Experience Sampling Method. PLoS One 2016; 11: e0151195.

6. Schenkman M, Ellis T, Christiansen C, et al. Profile of functional limitations and task performance among people with early- and middle-stage Parkinson disease. Phys Ther 2011; 91: 1339–1354.

7. Mancini M, El-Gohary M, Pearson S, et al. Continuous monitoring of turning in Parkinson's disease: Rehabilitation potential. NeuroRehabilitation 2015; 37: 3–10.

8. Mayagoitia RE, Nene AV, Veltink PH. Accelerometer and rate gyroscope measurement of kinematics: an inexpensive alternative to optical motion analysis systems. J Biomech 2002; 35: 537–542.

9. Seimetz C, Tan D, Katayama R, Lockhart T. A comparison between methods of measuring postrual stability: force plates versus accelerometers. Biomed Sci Instrum 2012; 48: 386–392.

10. Kassavetis P, Saifee TA, Roussos G, et al. Developing a tool for remote digital assessment of Parkinson's Disease. Movement Disorders Clinical Practice 2016; 3: 59–64.

11. White DK, Wagenaar RC, Del Olmo ME, Ellis TD. Test-retest reliability of 24 hours of activity monitoring in individuals with Parkinson's disease in home and community. Neurorehabil Neural Repair 2007; 21: 327-340.

12. Horak FB, Mancini M. Objective biomarkers of balance and gait for Parkinson's disease using body-worn sensors. Mov Disord 2013; 28: 1544–1551.

13. Mancini M, Carlson-Kuhta P, Zampieri C, Nutt JG, Chiari L, Horak FB. Postural sway as a marker of progression in Parkinson's disease: a pilot longitudinal study. Gait Posture 2012; 36: 471–476.

14. El-Gohary M, Pearson S, McNames J, et al. Continuous monitoring of turning in patients with movement disability. Sensors (Basel) 2013; 14: 356–369.

15. Terashi H, Mitoma H, Yoneyama M, Aizawa H. Relationship between Amount of Daily Movement Measured by a Triaxial Accelerometer and Motor Symptoms in Patients with Parkinson’s Disease. Applied Sciences 2017; 7: 486.

16. Motl RW, McAuley E. Pathways between physical activity and quality of life in adults with multiple sclerosis. Health Psychol 2009; 28: 682–689.

17. Kapoor T, Sharma RK. Parkinson’s disease diagnosis using Mel-frequency cepstral coefficients and vector quantization. Int J Computer Appl 2011; 14: 43–46.

18. Salarian A, Zampieri C, Horak FB, Carlson-Kuhta P, Nutt JG, Aminian K. Analyzing 180 degrees turns using an inertial system reveals early signs of progression of Parkinson's disease. Conf Proc IEEE Eng Med Biol Soc 2009; 2009: 224–227.

19. Cheng WY, Scotland A, Lipsmeier F, et al. Human activity recognition from sensor-based large-scale continuous monitoring of Parkinson’s disease patients. 2017 IEEE/ACM International Conference on Connected Health: Applications, Systems and Engineering Technologies (CHASE); Philadelphia, PA2017.

20. Haubenberger D, Abbruzzese G, Bain PG, et al. Transducer-based evaluation of tremor. Mov Disord 2016; 31: 1327–1336.
